# The Future of Paediatric Heart Interventions: Where Will We Be in 2030?

**DOI:** 10.1007/s11886-020-01404-z

**Published:** 2020-10-09

**Authors:** Tomohito Kogure, Shakeel A. Qureshi

**Affiliations:** 1 Department of Congenital Cardiology, Evelina London Children’s Hospital, Guy’s and St Thomas’ NHS Foundation Trust, London, SE1 7EH UK; 2 Department of Cardiology, Tokyo Women’s Medical University, Tokyo, 162-0054 Japan

**Keywords:** Congenital heart disease, Cardiac catheterization, Biodegradable devices, Percutaneous pulmonary valve implantation, MRI-guided interventions

## Abstract

**Purpose of Review:**

Cardiac catheterization therapies to treat or palliate infants, children and adults with congenital heart disease have developed rapidly worldwide in both technical innovation and device development in the previous three decades. By reviewing of current status of novel or development of devices and techniques, we will discuss what is likely to happen in paediatric heart intervention in the next decade.

**Recent Findings:**

Recently, biodegradable stents and devices, transcatheter pulmonary valve implantation for the native right ventricle outflow tract and MRI-guided interventions have been progressing rapidly with good immediate to early results. These are expected to be introduced and spread in the next decade although there are still challenges to overcome.

**Summary:**

The future of paediatric heart intervention is very promising with rapid development of technological progress.

## Introduction

Cardiac catheterization therapies for infants, children and adults with congenital heart disease (CHD) have progressed rapidly worldwide since Dr. William Rashkind reported the first balloon atrial septostomy for transposition of the great arteries in 1966 [1]. This was followed by the development of balloon valvoplasties and angioplasties in the 1980s [2, 3]. Stent implantation and coil embolization were developed in the 1990s [4, 5], and device closure of septal defect and patent ductus arteriosus became popular throughout the world in the 1990s and 2000s [6–9], as well as development of imaging technology [10, 11]. Nowadays, hybrid interventions [9, 12] and percutaneous pulmonary valve implantation are performed, supported by three-dimensional echocardiography or CT or MRI. Catheter intervention has become well-established, and the therapeutic range of interventions continues to expand with development of newer devices and techniques. In this article, we will describe the current status and future expectation of biodegradable stents and devices, transcatheter pulmonary valve implantation for the native right ventricle outflow tract and MRI-guided interventions as these are expected to become widespread in the next decade.

## Biodegradable Stents and Devices

### Biodegradable Stents

For congenital heart disease, biodegradable stents will likely have the most utility in small infants and children. Implantation of balloon-expandable metal stents is the primary technical tool to treat vascular stenosis. The major limiting factors using bare metallic stents for paediatric field are lack of growth as the child grows, and limited expansion potential, which might require complicated surgical reintervention, in addition to other common complications, such as stent thrombosis and late in-stent stenosis. The concept of biodegradable stent (BDS) has found traction as an alternative to bare metallic stents, both in adult and paediatric applications. BDS has a number of advantages over existing bare metallic stent designs. Once biodegraded, they potentially leave behind a healed endothelialized natural vessel with further growth potential. Furthermore, BDS can act as a platform similar to their metal counterparts for either drugs or genes to promote vessel growth. Finally, BDS is readily compatible with MRI and CT imaging.

Preclinical and clinical coronary studies showed late positive remodeling with normal vasomotor function after the stent disappears. BDSs have the potential to eliminate the need for repeated interventions. Long-term animal studies are warranted to confirm late positive remodeling and evaluate vessel growth and function following complete stent degradation and assess risks associated with stent fragment embolization during the degradation process.

Case reports using coronary artery bioresorbable stents in paediatric patients with small vessels off-label in Europe have emerged. Zartner et al. [13] have described the use of the Biotronik stent (Biotronik, Berlin, Germany), a magnesium stent, for stenting of a stenotic left pulmonary artery in a preterm infant. The same group also was the first to report the use of the stent for the treatment of an infant with recoarctation [14]. The bioresorbable vascular scaffold (BVS) system (Abbott Vascular, Santa Clara, CA, USA) had been commercially available, and some congenital centres attempted to use the BVS for infants with pulmonary arterial and venous disease [15]. Despite encouraging case reports for congenital heart disease, the Biotronik stent and BVS were discontinued by the companies because of disappointing long-term results for the treatment of coronary disease [16]. Currently, some of the different groups across the world have been working towards producing a BDS for paediatric patients with CHD [17]. The Illusicor stent (Tremedics Medical Devices LLC, TX, USA) and 480 Biomedical stent (480 Biomedical Inc., MA, USA) have been developed as a bioresorbable vascular scaffold (BRS) for CHD with preclinical experience [18–20]. Zinc bioresorbable stent (ZeBRa stent) (PediaStent LLC, OH, USA) is the newest BDS specifically designed for CHD. Table 1 depicts the current BRS for paediatric application still under development. In coronary interventions, the current study provides insights into the safety and performance of the MeRes100 stent (Meril Life Sciences Pvt. Ltd., Vapi, India), a novel second-generation sirolimus-eluting BRS, with up to 3-year follow-up [21]. Another second-generation BRS named Magmaris (Biotronik AG, Bülach, Switzerland), a newer generation magnesium-based BRS, has reported encouraging results based on a small number of non-RCT studies [22], and one case was reported as the first use of a Magmaris for native aortic coarctation in a small infant with success as a short-term bridge-to-surgery [23].

**Table 1:** Current biodegradable stents for paediatric application

Stent	Company	Material	Design	Size variation	Profile	Reabsorption (months)	Approval stage
Illusicor stent	Tremedics Medical Devices LLC	PLLA*	Balloon expandable	3–8 mm diameter (ongoing evaluation for 10–20 mm)	5 or 6-Fr delivery	24 months	Preclinical (ref 18, 19)
480 Biomedical stent	480 Biomedical Inc.	PLGA^†^/PLCL^‡^	Self-expanding	7–10 mm diameter	5 or 6-Fr delivery	12–18 months	Preclinical (unpublished data)
ZeBRa stent	PediaStent LLC	Zinc alloy	Balloon expandable	6 mm diameter	N/A	N/A	Preclinical (unpublished data)

*PLLA poly l-lactic acid, ^†^*PLGA* poly lactic-co-glycolic acid, ^‡^*PLCL* poly lactide-co-ε-caprolactone

### Biodegradable Devices

Transcatheter closure is the current standard of care for the treatment of atrial septal defects worldwide. The current devices utilize a metal framework to support the occlusive material and to permanently clamp the septum. The long-term presence of a significant amount of metal in the heart leads to rare but serious complications such as erosion, arrhythmias and thrombus formation, most likely related to incomplete endothelialization [24–28]. In addition, it makes crossing of the septum to access the left side of the heart difficult for future interventions, which significantly reduces future treatment options for these patients. Hence, biodegradable occluders have become a desirable alternative requirement. As early as 2003, the first partially degradable occluder, BioSTAR (NMT Medical, Boston, MA, USA) with a metallic permanent framework (MP35N) and a bioresorbable membrane made from intestinal collagen, was introduced [29–33]. A tremendous step forward from non-degradable metal occluders to biodegradable occluders was made based on the successful development of BioSTAR and desirable clinical results, even though it was withdrawn from the market due to complications with wire fractures and inflammatory reactions locally related to the biologic material of the membrane [33]. Currently, several partially or fully biodegradable occlusion devices are being developed and evaluated [34–42] and have been at varying stages of animal experimental or clinical trial (Table 2). The Carag bioresorbable septal occluder (CARAG AG, Baar, Switzerland) is the only device that is CE (Conformité Européene) marked.

**Table 2:** Partially or fully biodegradable occlusion devices

Device	Year of introduction	Company or institute	Design	Biodegradability	Human study	Ref. no.
Immediate release patch	2002	Custom Medical Devices, Greece	Sleeve-shaped patch made of made from biodegradable polyurethane foam	Fully	Very small multicentre study (*n* = 13)	37
Carag bioresorbable septal occluder	2014	CARAG AG, Switzerland	Round double disc made of bioresorbable PLGA* with two opposing foldable polyester covers	Partially	First-in-human study (*n* = 14)	46, 47
Double BioDisk	2010	COOK Medical, USA	Porcine small intestinal submucosa covered disc with two flexible nitinol rings	Partially	Animal study only	38
Double-umbrella occluder	2010	Nanyang Technological University, Singapore	Two self-expanding umbrellas disc made of PLC**	Fully	Animal study only	39
Chinese Lantern occluder	2011	Nanyang Technological University, Singapore	Fully biodegradable polymers (PLC and PCL***) featured with a unique pull-fold mechanism	Fully	Animal study only	40
Fully biodegradable ASD occluder	2012	Second Military Medical University, China	Double-disc device made of polydioxanone which is similar design to Amplatzer septal occluder	Fully	Animal study only	41
Totally biodegradable PLA-based occluder	2018	Shanghai Shape Memory Alloy Co. Ltd. China	PLLA^†^ skeleton and two discs made of PDLLA^‡^ fabrics	Fully	Animal study only	42
Fully degradable PLLA occluders	2016	Guangdong Academy of Medical Sciences, China	Double-umbrella framework and two battle fabrics both composed of PLLA^†^	Fully	Animal study only	43

*PLGA poly lactic-co-glycolic acid, **PLC poly lactide-co-ε-caprolactone, ***PCL poly ε-caprolactone, ^†^*PLLA* poly l-lactic acid, ^‡^*PDLLA* poly d l-lactic acid

#### Carag Bioresorbable Septal Occluder

The Carag bioresorbable septal occluder (CBSO) (CARAG AG, Baar, Switzerland) is a self-centering, double-disc device without any metal framework, composed of poly lactic-co-glycolic acid. Endothelialization of the device seems to be completed within 3 months, while the device usually starts to be resorbed after 6 months and completely resorbed within 2 years. CBSO has 3 size options: small for 4–12-mm defects, intermediate for 13–20 mm, and large for 21–25-mm defects. The animal trials were completed in 24 German Landrace pigs with created interatrial septal defects. All occlusion devices were observed to be correctly positioned without any residual shunt or dislocation after implantation, and there were no procedural or device-related complications. Complete endothelializations covered by thin and smooth whitish tissue layers were achieved in all specimens. No cellular inflammation reaction was observed [43•]. The CBSO was implanted in a first-in-man in 2014 in Germany and completed CE marking in 2017 as a result of preliminary data of successful outcome in all 14 patients, 8 with ASD and 6 with patent foramen ovale [44, 45]. Several centres in Germany, Switzerland and other European countries will participate in the CBSO registry, post CE mark study, which is aiming to enroll up to 100 patients. Carag intends to perform a clinical trial in the USA under an investigational device exemption (IDE).

### Future Perspectives

Biodegradable stents have additional challenges to overcome. Infants and children with CHD often need larger diameter stents to dilate obstructed blood vessels such as the aorta and pulmonary arteries. There are significant biomedical and engineering challenges related to durability from repeat collapse pressure and increasing elastic recoil with manufacture of larger diameter stents, especially when constructed from polymers. As ideal absorption time is unknown, there are ongoing concerns for morbidity related to thromboembolism of the BDS fragments to distal vasculature and local reactions to the degrading stents. However, there is a huge need for BDSs, which are desirable in children.

Biodegradable devices are a promising future. Some devices will be introduced into the market and may be able to replace metal devices. It makes it realistic to use the devices for small children and patients with severe metal allergies, who have been hesitant to be treated percutaneously. Furthermore, it is expected that similar techniques will be applied to other devices, such as patent ductus arteriosus occluder, ventricular septal defect occluder or other plug devices.

## Transcatheter Pulmonary Valve Implantation for the Native Right Ventricular Outflow Tract

Pulmonary regurgitation after surgical repair of tetralogy of Fallot (ToF) is almost inevitable and, in the past, has required repeat surgery for replacing the regurgitant pulmonary valve. After 2000, percutaneous pulmonary valve implantation (PPVI) with balloon-expandable valves has replaced the surgical approach in a select group of patients. However, PPVI with the commercially available balloon-expandable valves have limitations because of their size, and being applicable to right ventricle-pulmonary artery conduits or bioprosthetic valves [46, 47]. They have been considered unsuitable for native dilated right ventricular outflow tracts (RVOTs) after a transannular patch repair technique, which form about 70–75% of the patients [48–52]. Novel self-expanding pulmonary valves, the Venus P-valve (Venus Medtech, Shanghai, China), the Pulsta valve (TaeWoong Medical Co., Ltd., Gimpo-si, Gyeonggi-do, South Korea) and the Harmony transcatheter pulmonary valve (Medtronic, Minneapolis, MN, USA), have been developed for this specific patient population.

**Fig. 1:**
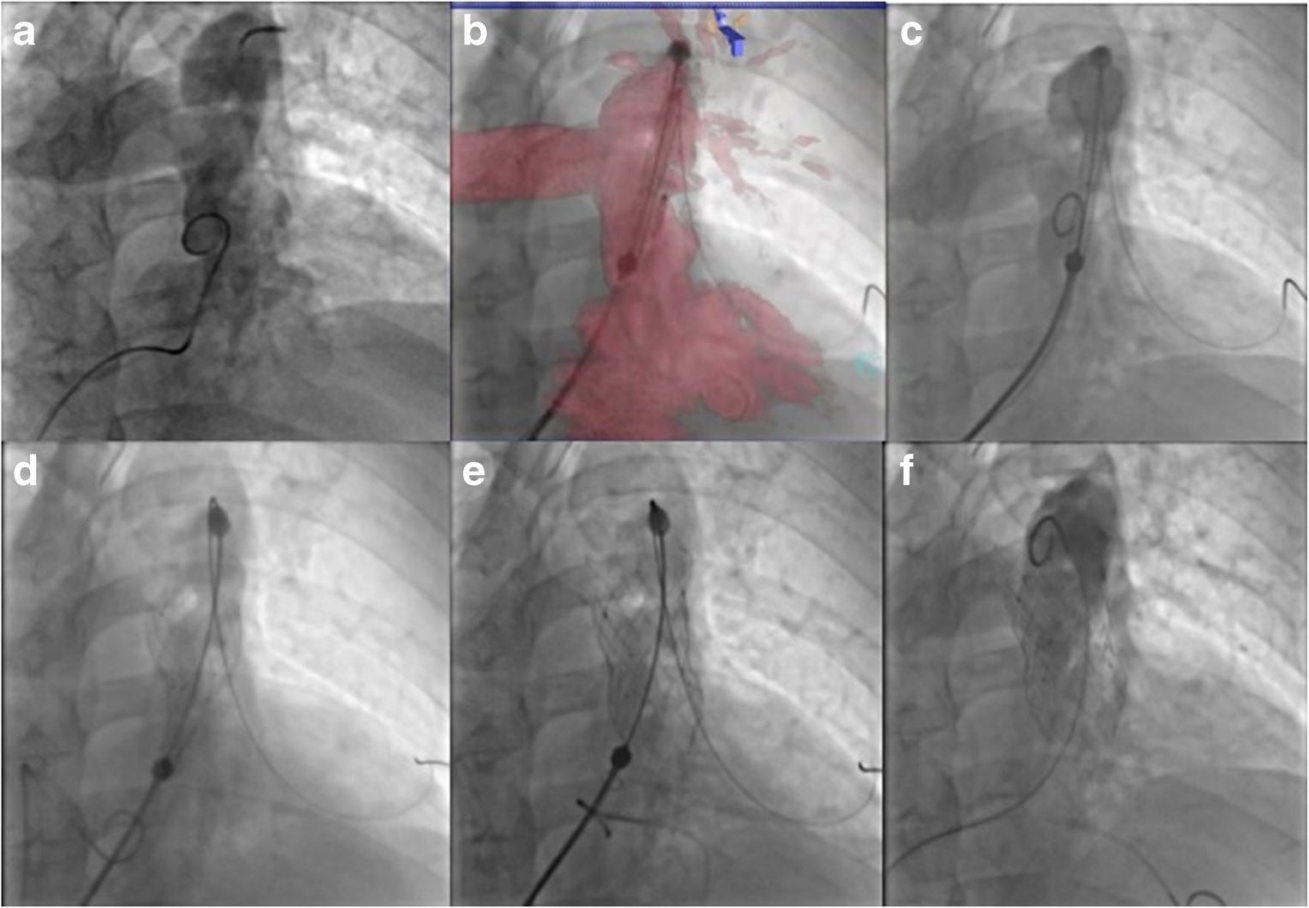
Deployment of the flared Venus P-valve. **a** Angiogram in the main pulmonary artery showing severe pulmonary regurgitation. **b** Positioning of the distal carrot of the Venus P-valve in the left pulmonary artery (LPA) using fusion imaging. **c** The deployment starting position in the proximal LPA. **d**, **e** After exposing the distal flare, the system is pulled free from the LPA origin before gradually deploying the rest of the valve frame. **f** Final angiogram after Venus P-valve implantation showing no pulmonary regurgitation

All three valves are a porcine pericardial tissue valve mounted on a self-expanding nitinol frame and have proximal and distal flares that anchor the valve in the right ventricular outflow tract. The Venus P-valve is one of the leading valves, which has an asymmetrical covering design, in which the pulmonary artery end flare is not covered, permitting unobstructed flow into the branch pulmonary arteries. Currently, the Venus P-valve is available in diameters from 16 to 36 mm with 2-mm increments, with each diameter available in 20-, 25-, 30- and 35-mm straight section lengths (Fig. 1). The valve can be implanted in the RVOTs, whose narrowest diameter is up to 33–34 mm, for which a 36-mm-diameter valve can be implanted, which is the largest diameter valve currently available [53, 54••].

The Harmony transcatheter pulmonary valve is the only valve that will be available in the USA in the near future. The device has an outer diameter of 23.5 mm at the valved section and is approximately 55 mm in length. Lately, 3-year outcomes of Food and Drug Administration–approved early feasibility study have been reported by Benson et al. with a promising result of stable device position, good valve function in most, and the absence of moderate/severe paravalvular leak and significant late frame fractures [55]. Table 3 summarizes clinical studies of the commercially approved valves and the new self-expanding valves reported in the literature. Early experience with these valves, although limited, has been encouraging, suggesting that self-expandable pulmonary valves can be implanted safely in patients with native RVOTs, with good short- and mid-term results [55–62].

**Table 3:** Summary of clinical studies in percutaneous pulmonary valve implantation

Valve	First author, year (ref. no.)	Country	Time period	*N*	Age (years)	Weight (kg)	Follow-up (months)	Mean valve size	Implantation success* (%)	Complications					Unplanned intervention	Mortality (%)
										Valve migration	RVOT rupture	Stent fracture (SF-r**)	Infective endocarditis	Moderate PS or PR		
Melody	McElhinney et al. 2010 (48)	USA	2007 to 2009	30	19	61	12	NA	29 (96.6%)	0	1	8 (3)	0	5	Surgical 1 Catheter 4	0
SAPIEN	Kenny et al. 2011 (49)	USA, UK	2008 to 2010	36	30	73	6	NA	35 (97.2%)	3	0	0	0	1	Surgical 4 Catheter 1	0
Venus P	Morgan et al. 2019 (56)	UK, others	2013 to 2017	38	24	59	25	NA	36 (94.7%)	2	0	8 (0)	0	0	Surgical 1	0
Pulsta	Kim et al. 2018 (59)	South Korea	NA	10	21	59	6	27.0 mm	10 (100%)	0	0	0	0	0	0	0
Harmony	Benson et al. 2020 (64)	USA, Canada	2013 to 2015	20	28	72	36	23.5 mm	20 (100%)	2	0	4 (1)	0	3	Surgical 2 Catheter 2	0

*Defined as the percentage of subjects with a transcatheter pulmonary valve placed with no more than mild PR, an RV-PA peak-to-peak gradient < 35 mmHg by angiography

**SF-r stent fractures requiring reintervention

The Med-Zenith PT-Valve (Med Zenith, Beijing, China) is another self-expandable valve with good immediate result of first human study [63]. The Alterra Adaptive Prestent is another new concept of a self-expanding, partially covered stent, which was designed to internally reconfigure the native dilated RVOTs, such as to make them suitable for implantation of a commercially available balloon-expandable heart valve, the SAPIEN S3 transcatheter heart valve (Edwards Lifesciences, Irvine, CA, USA). The device has a symmetrical frame design to provide a rigid “landing zone” for a SAPIEN S3 (29 mm). Zahn et al. reported the first human implantation, which has shown encouraging results [64].

### Future Perspectives

With further developments in the technology, a significant majority of patients may be potentially treatable by transcatheter valve technology within the next decade. The number of patients in whom transcatheter valve therapy may be an option has expanded with the advent of novel valves designed specifically for use in the larger, non-conduit, outflow tracts. Once this technology and valve-in-valve strategy are established, it will be possible to avoid repeat midline sternotomy and reduce the risk of repeat open surgery for the majority of ToF patients. In the not too distant future, if designing a percutaneous valve made of large scaffold with leaflets made of stem cell technology becomes a reality, it may be possible to avoid reoperations.

## MRI-Guided Interventions

Diagnostic and interventional cardiac catheterization is routinely used in the diagnosis and treatment of CHD. However, the use of radiation during repetitive x-ray–guided catheterization has led to concerns relating to the risk of solid tumours in later life, and that is particularly true in children, in whom increased radiation sensitivity, coupled with the possibility of repetitive exposure to diagnostic and interventional x-ray procedures, can lead to a significant increase of cancer risk [65–70]. Magnetic resonance imaging (MRI) not only avoids exposure to radiation but also has the advantage of being able to provide better soft tissue visualization, tissue characterization and quantification of ventricular volumes and vascular flow [71, 72].

Initial work using MRI catheterization employed fusion of x-ray and MRI techniques, with x-ray fluoroscopy to guide catheter placement and subsequent MRI for anatomical and hemodynamic assessment. Image overlay of previously acquired 3D MRI datasets with live fluoroscopic imaging has also been used to guide catheter procedures [10]. More recent developments in passive and active catheter tracking have led to improved visualization of catheters for MRI-guided catheterization [73, 74]. Continuous development of equipment has also increased image quality and scanning times with better interactive tools for the operator in the MRI catheter suite to navigate through the anatomy as required in real time [75]. This has expanded to MRI-guided electrophysiology (EP) studies and radiofrequency ablation in humans [76]. Animal studies show promise for the utility of MRI-guided interventional catheterization. With ongoing investment and development of MRI-compatible guidewires, certain percutaneous cardiac interventions may be performed solely under MR guidance in the future [77, 78].

### Interventional Congenital Cardiac Procedures

There have been a number of animal studies showing the safety and efficacy of interventional cardiovascular magnetic resonance (CMR) for congenital cardiac procedures. Animal interventional CMR has been used to facilitate procedures such as balloon angioplasty of arterial stenosis [79–81], atrial septal puncture/septostomy [82] as well stenting of pulmonary arteries [83], aortic coarctation [84, 85] and vena cava intervention [86, 87]. Device closure of atrial septal defects is another application that has been explored [88–90]. Further animal studies also offer potential interventions in CHD such as transcatheter implantation of a prosthetic valve [91], percutaneous ventricular septal defect closure [92] and transcatheter creation of cavopulmonary shunts [93, 94•]. Based on encouraging preclinical studies, the application of interventions has now been extended to humans. We reported the first-in-man solely MR-guided CHD interventions, balloon dilation of pulmonary valve, with promising results [95], and Krueger et al. reported balloon dilation of aortic coarctation under CMR guidance alone [81].

### Future Perspectives

**Fig. 2:**
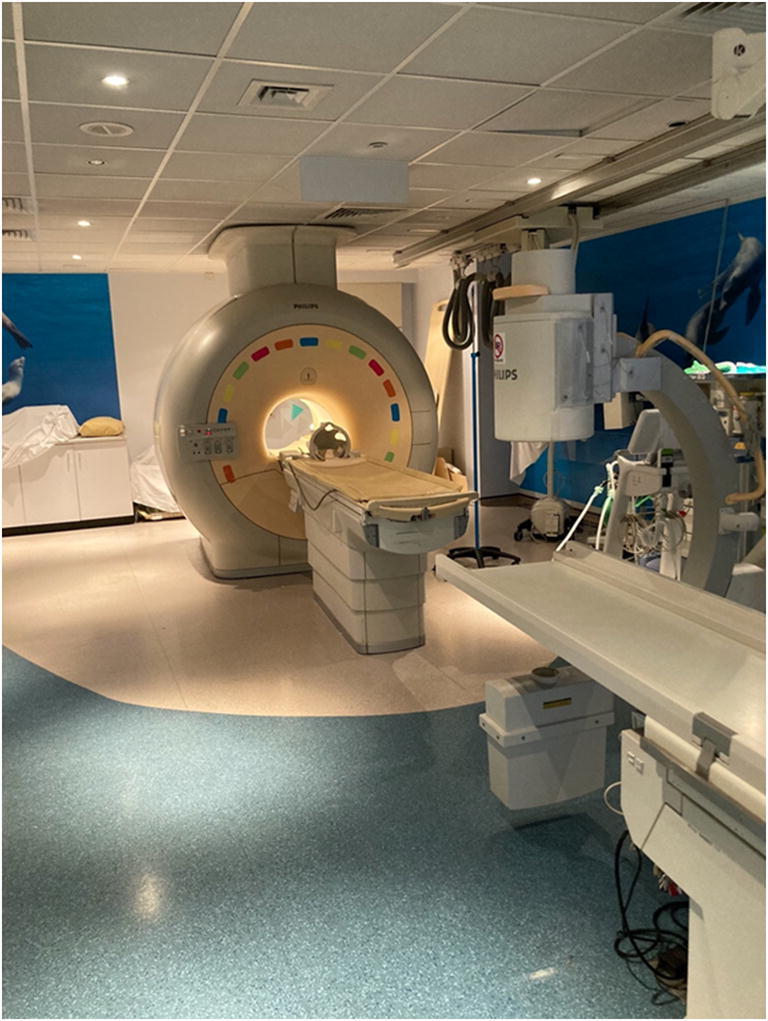
X-ray and CMR (XMR) room. XMR room with the x-ray and MR equipment joined by a movable tabletop. The c-arm of the x-ray unit is seen in the right side and the 5-gauss area is demarcated by a change in the floor colouring from the MR to the x-ray end of the room

MR imaging–guided cardiac catheterization has become a clinical reality (Fig. 2), after years of technological development and preclinical/clinical testing [11, 71, 96]. There is a move for industry participation in the development of CMR-compatible cardiac catheters and devices specifically designed for CMR-guided cardiac intervention. Cardiac EP is the first field for which clinical grade device development is complete and in which human clinical trials are currently underway. Regarding congenital heart field, complex anatomy particularly requires wires and end-hole catheters with good steerability and torque to negotiate the bends of the relevant cardiac and vascular structures. Development needs to keep pace with the meticulous processes of regulatory approval of devices and also needs to be some verification in terms of the cost-effectiveness of these techniques and their role in improving patient outcomes. However, CMR-guided interventions in paediatric cardiology will continue to develop as a consequence of the continued striving for better anatomical and physiological data and avoidance of radiation. We predict that the next decade will see interventional MRI catheterization become a more widespread clinical reality.

## Conclusions

Paediatric heart intervention has progressed from the initial balloon atrial septostomy performed in the 1960s. The future of interventions is very promising and exciting with the development of devices, techniques and imaging modalities. Bioresorbable stents and devices may be available commercially for use in CHD, and more patients will benefit from transcatheter pulmonary valve implantation. MRI-guided interventions in children will be introduced for wider clinical use in not too distant future. Technological progress is occurring at a tremendous pace. Innovative technology such as stem cell techniques and newer device development will be applied for treatment in paediatric cardiology.
